# Early-Stage Primary Rectal Melanoma: A Case Report

**DOI:** 10.7759/cureus.42629

**Published:** 2023-07-28

**Authors:** Zahira El Youssi, Hanane Mansouri, Sofia Elouaouch, Mohammed Moukhlissi, Soufiane Berhili, Loubna Mezouar

**Affiliations:** 1 Radiation Oncology, Mohammed VI University Hospital, Faculty of Medicine and Pharmacy, Mohammed I University of Oujda, Oujda, MAR

**Keywords:** mucosal malignant melanoma, rectum, radiotherapy, treatment, pronostic

## Abstract

Malignant primary rectal melanomas (PRM) are rare tumors. Their diagnosis is frequently delayed as these lesions are often mistaken for benign diseases, resulting in extremely poor overall survival. Histological evaluation with special immunohistochemical (IHC) stains is often indispensable for a definitive diagnosis. The main treatment for this condition involves surgical resection. Adjuvant therapy has also been long recommended. We discuss the case of a 60-year-old woman who presented with changes in bowel habits, anal pain, and perineal burning with no bleeding. A digital rectal examination revealed a nodular mass extending 5 cm from the anal verge. Rectosigmoidoscopy demonstrated an ulcerated polypoid tumor extending 4 cm from the anal verge and over 5 cm into the lower rectum. Biopsy and IHC tests confirmed the diagnosis of rectal melanoma. The patient was successfully managed with surgery followed by external beam radiotherapy and a complete response was achieved after 10 months of follow-up.

## Introduction

Since mucosal melanomas (MMs) account for less than 1% of all cases of melanoma, this rare entity can develop on any mucosal surface due to the proliferation of pigment-producing melanocytes. However, the head and neck account for the majority of cases followed by the anorectum, which represents 24% of cases [1,2]. Anorectal malignant melanoma (ARMM) is an aggressive but uncommon subtype that represents only 0.05% of all malignancies arising in the anorectal area [3]. It most commonly occurs in female patients between the fifth and eighth decades of life (54-76%) [4]. Its etiology and pathogenesis are still poorly understood.

Given its anatomic location and nonspecific symptoms, which are frequently confused with other conditions, ARMM can be quite challenging to diagnose at early stages, leading to poor survival rates [1]. Due to its rarity, the optimal management of this condition remains a matter of controversy. While the mainstay of treatment is surgical removal, other treatments such as radiation therapy and chemotherapy are considered limited options despite their efficiency [5].

We present a case of a 60-year-old female diagnosed with primary rectal melanoma (PRM) who was managed with wide local excision (WLE) followed by adjuvant radiotherapy.

## Case presentation

**Figure 1:**
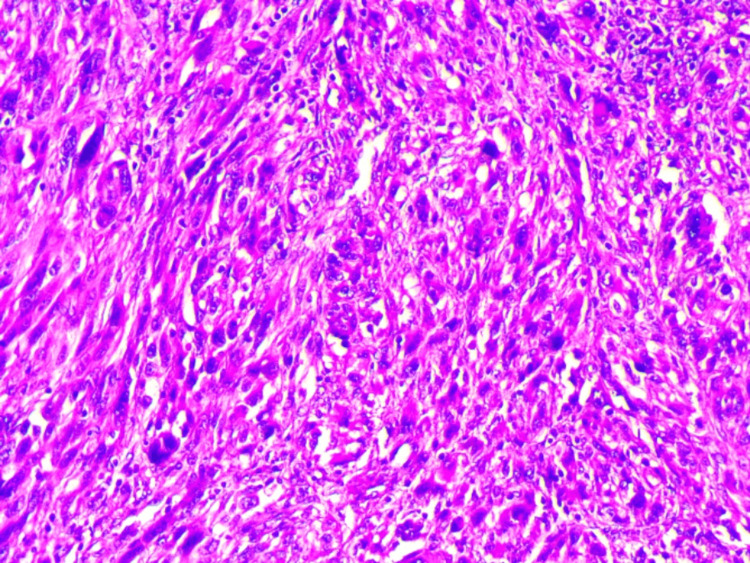
Pathology findings (H&E, x400) Tumor cells are fusiform, with elongated nuclei and mottled chromatin. Numerous mitotic figures are seen

A 60-year-old female was admitted with a one-month history of changes in bowel habits, anal pain, and perineal burning with no bleeding. The patient had been a known diabetic for 20 years with high blood pressure and had been under treatment; her family history was negative for colon and/or rectal carcinoma. The abdominal examination was unremarkable. A digital rectal examination revealed a nodular mass extending 5 cm from the anal verge. A full colonoscopy demonstrated an ulcerated polypoid tumor extending over 5 cm, and 4 cm from the anal verge, and involving half the circumference; no synchronous lesions were detected. Histopathological examination (HPE) of the mass revealed nests of heavily pigmented fusiform malignant cells containing melanin pigment, and the diagnosis of malignant melanoma was considered (Figure 1).

**Figure 2:**
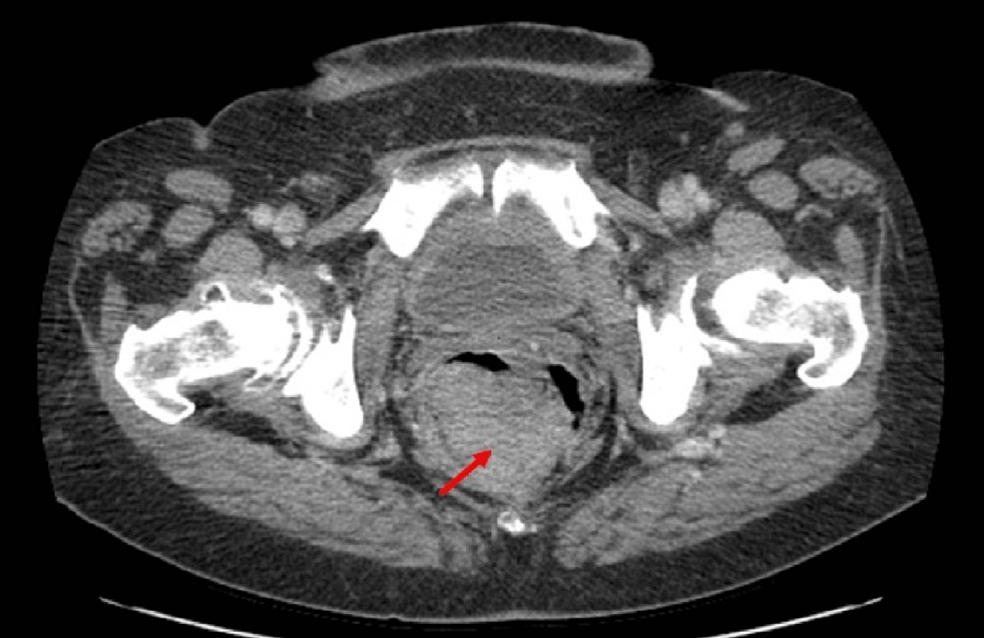
Axial contrast CT of the pelvis showing an irregular and heterogeneous rectal tumor (arrow) CT: computed tomography

According to immunohistochemical (IHC) staining, the melanoma markers S-100, Melan-A, and HMB-45 were positive. There was no history of other symptoms or a pigmented skin lesion to suggest another possible primary site. Molecular biology showed a TP53 mutation but no B-type Raf (BRAF) or receptor tyrosine kinase (c-KIT) mutations. Thoracic abdominal pelvic contrast CT revealed a large, irregular, heterogenous rectal mass measuring 5.8 x 5 cm and no evidence of nodal or distant metastases (Figure 2).

The patient proceeded to have surgery and underwent WLE; the recovery was unremarkable. Histologically, the sections showed a widely ulcerated anorectal mucosa infiltrated by a melanic process, with infiltration of the submucosa and the presence of perineural invasion. Adjuvant external beam radiotherapy was recommended at a total dose of 48 Gy in 20 fractions of 2.4 Gy, and no other adjuvant therapy was indicated. The patient was seen for a follow-up 10 months after undergoing the procedure. No residual tumor was found on digital rectal and sigmoidoscopic examinations, and the CT scan of the chest, abdomen, and pelvis was clear.

## Discussion

Primary MMs were identified for the first time in 1859 by Weber [1]; this entity can be classified based on the anatomic location of the tumor, and the anorectum is the second most common subsite of MMs [6]. ARMM is extremely rare, representing less than 1% of all melanomas, between 1% and 2% of lower GI malignant tumors, and 0.1% of all rectal cancers [7,8]. This makes rectal melanomas exceedingly uncommon. PRM is a malignant tumor arising from melanocytes in the rectal mucosa and located over 4 cm from the anal verge [9]. The literature suggests that immunology has an impact on the development of rectal melanoma. Patients with human papillomavirus (HPV) and human immunodeficiency virus (HIV) infections appear to have a higher risk, but there is no definitive evidence to support this [5].

MM differs from cutaneous melanoma in numerous aspects. The pathogenesis of this tumor is generally unknown. According to some reports, this neoplasm has many genetic alterations in intracellular signaling cascades, and the ideal approach to target it is yet unknown [10]. In addition, tyrosine kinase (KIT) mutations were initially identified in MMs by Curtin et al. [10] and were reported in 35.5% of anorectal melanomas [11,3]. However, mutations in BRAF are infrequent and seen in fewer than 10% of all MMs [10,12]. MMs generally have been reported to occur in the fifth to eighth decades of life, with a median age of around 60-70 years [1], and it appears most commonly in females (58.1-63.1%) [4,13].

Given its nonspecific symptoms, ARMM might be challenging to identify. Diagnosis and early treatment are often delayed because these tumors are often confused with benign anorectal diseases such as hemorrhoids and rectal polyps [5,12]. Rectal bleeding is the most frequent symptom at presentation (54-78%). A palpable mass (34%), incontinence, pain, and a change in bowel habits as seen in our case are among the other symptoms [1,4]. The rectal digital examination allows to evaluate the ulceration, size, and fixation of the tumor. Rectosigmoidoscopy may enable the diagnosis of anorectal melanoma if melanin pigmentation is clearly detected [4].

A full colonoscopy is recommended to rule out synchronous lesions and for tissue biopsy. Endorectal ultrasonography, CT, and MRI may be useful in determining tumor size, the existence of regional lymph node metastases, and for systemic staging. Approximately, 61% of patients with ARMM present with regional nodal involvement [1], and 26-38% of patients already have metastasis at the time of diagnosis [12]. Our patient had no evidence of nodal or distant metastasis at the presentation on the CT scan. HPE is necessary for rectal melanoma diagnosis. This tumor can mimic other malignant tumors, such as small round cell sarcoma and epidermoid carcinoma [4]. It is based on the detection of the melanin pigment within the tumor. Moreover, IHC staining can be necessary in atypical forms, using anti-Melan-A and anti-HMB-45 antibodies, which were positive in our case.

Notwithstanding their rarity, MMs have been of significant concern due to their aggressive nature and have a poorer prognosis when compared with cutaneous melanomas, and like most malignancies, early detection is critical for optimizing the chances of survival [10,2]. Many prognostic factors have been identified in the literature. Some authors believe that the prognosis of anorectal melanoma, in general, is completely determined by the stage of the tumor at the time of diagnosis, whether local, regional, or systemic. The presence of perineural invasion, tumor necrosis, infiltration depth of the rectal wall, lymph node metastasis, and distant metastases have been associated with poor overall survival [14,5]. However, it has been shown that survival rates improved when tumor thickness was less than 4 mm [15]. Due to the delayed presentation and diagnosis resulting in an advanced-stage disease at the time of the diagnosis, these patients often have a five-year survival rate of less than 20% [7].

in view of the rarity of the disease and the lack of randomized controlled trials to devise an optimal treatment, surgery remains the major therapeutic intervention for rectal melanomas [2,4]. Abdominal perineal resection (APR) and WLE are surgical techniques that can be employed [4]. In order to minimize morbidity and when it is technically feasible, WLE seems to be the treatment of choice for a curative approach [5,16]. It is recommended for patients in the early stages of the disease such as our case, small tumors, and those with no evidence of nodal metastases [4,5]. Several retrospective studies have found that there is no significant difference in survival between WLE and APR [5,17], and no evidence of survival benefit has been attributed to any particular type of resection [2,18]. Nevertheless, tumor depth is the strongest predictor, which enables making the decision between WLE and APR. Thus, for tumor thickness above 4 mm, APR should be indicated [5,15].

Currently, no adjuvant therapy can be considered the standard of care for the treatment of anorectal melanoma. In general, radiotherapy has a limited role in MM. It may be used to achieve local control in patients who are unable to undergo surgical resection, or to improve control after surgery, which is the purpose of the treatment, particularly when resection margins are insufficient. As in our patient, adjuvant radiotherapy has been shown to be effective as a complementary method to WLE in anorectal melanomas, as an alternative to APR in terms of quality of life and decreasing local recurrence [2,4]. For palliative purposes, radiation can be useful to control tumor bleeding [12]. Many other adjuvant therapeutic options have been developed, and a better comprehension of the molecular aberrations of MM has revealed significant information on the targeted therapies. Tumors with KIT anomalies have a better response to KIT inhibitors. In some studies, it has been shown that tumors with particular KIT alterations as ”mutations” may have more susceptibility to respond to these molecules than others [2]; as a result, a selection based on molecular screening is crucial to identify patients who might potentially benefit [19].

There is currently no standard approach for systemic therapy treatment of resectable MM. Vemurafenib and dabrafenib were developed to inhibit BRAF V600 mutations; adjuvant dabrafenib/trametinib combination therapy is a recommended option for patients with resected stage III or recurrent disease and who harbor a BRAF V600-activating mutation. Based on the results of several studies, the PD-1-directed antibody (nivolumab) should be considered as an adjuvant postoperative treatment option for patients with stage III-IV disease at presentation [19]. For patients with metastatic disease, several phase III trials have shown that monotherapy with either vemurafenib or dabrafenib improves response rates and survival compared with chemotherapy. However, high-dose IL-2 has been used extensively to treat metastatic melanoma in first-line and second-line settings [19].

The recommended post-treatment surveillance program for patients following treatment for rectal melanoma includes external inspection, palpation of inguinal lymph nodes, digital examination, periodic evaluation by colonoscopy, as well as periodic chest, abdominal, and pelvic CT scans; CT or MRI of the brain at least once a year for life is recommended for all patients with melanoma [20].

## Conclusions

PRM is a rare but aggressive tumor with no pathognomonic signs. An early diagnosis is extremely crucial in managing these patients. Due to its rarity, adopting standardized treatment protocols has been challenging. The treatment should be focused on reducing morbidity and optimizing quality of life and function. Surgery remains the main treatment modality, while radiotherapy or systemic therapy are considered other options as adjuvant treatment. The prognosis seems to be bleak, despite all attempts to design a multimodal coordinated strategy.
